# Blood profile holds clues to role of infection in a premonitory state for idiopathic parkinsonism and of gastrointestinal infection in established disease

**DOI:** 10.1186/1757-4749-1-20

**Published:** 2009-11-26

**Authors:** André Charlett, R John Dobbs, Sylvia M Dobbs, Clive Weller, Mohammad AA Ibrahim, Tracy Dew, Roy Sherwood, Norman L Oxlade, J Malcolm Plant, James Bowthorpe, Andrew J Lawson, Alan Curry, Dale W Peterson, Ingvar T Bjarnason

**Affiliations:** 1 Therapeutics Research Group, Institute of Psychiatry at King's College London, London, UK; 2 Statistics Unit, Health Protection Agency, London, UK; 3 Department of Gastroenterology, King's College Hospital, London, UK; 4 Department of Immunology, King's College Hospital, London, UK; 5 Department of Biochemistry, King's College Hospital, London, UK; 6 Laboratory of Gastrointestinal Pathogens, Health Protection Agency, London, UK; 7 Electronmicroscopy, Health Protection Agency North West, Manchester, UK; 8 School of Life Sciences, University of Hertfordshire, Hatfield, UK

## Abstract

The two-stage neuroinflammatory process, containment and progression, proposed to underlie neurodegeneration may predicate on systemic inflammation arising from the gastrointestinal tract. *Helicobacter *infection has been described as one switch in the pathogenic-circuitry of idiopathic parkinsonism (IP): eradication modifies disease progression and marked deterioration accompanies eradication-failure. Moreover, serum *Helicobacter*-antibody-profile predicts presence, severity and progression of IP. Slow gastrointestinal-transit precedes IP-diagnosis and becomes increasingly-apparent after, predisposing to small-intestinal bacterial-overgrowth (SIBO). Although IP is well-described as a systemic illness with a long prodrome, there has been no comprehensive overview of the blood profile. Here, it is examined in relation to *Helicobacter *status and lactulose-hydrogen-breath-testing for SIBO.

A robust finding of reduced lymphocyte count in 126 IP-probands and 79 spouses (without clinically-definite IP), compared with that in 381 controls (p < 0.001 in each case), was not explained by *Helicobacter*-status or breath-hydrogen. This complements a previous report that spouses were 'down-the-pathway' to 'clinically-definite' disease. In 205 other controls without clinically-definite IP, there were strong associations between sporadic cardinal features and immunoglobulin class concentration, not explained by *Helicobacter*-status. Premonitory states for idiopathic parkinsonism associated with relative lymphopenia, higher serum immunoglobulin concentrations and evidence of enteric-nervous-system damage may prove viral in origin.

Although only 8% of the above 79 spouses were urea-breath-test-positive for *Helicobacter*, all 8 spouses with clinically-definite IP were (p < 0.0001). Transmission of a 'primer' to a *Helicobacter*-colonised recipient might result in progression to the diagnostic threshold.

Twenty-five percent of the 126 probands were seropositive for anti-nuclear autoantibody. In 20 probands, monitored before and serially after anti-*Helicobacter *therapy, seropositivity marked a severe hypokinetic response (p = 0.03). It may alert to continuing infection, even at low-density. Hyperhomocysteinemia is a risk factor for dementia and depression. Serum homocysteine exceeded the target in 43% of the 126 IP-probands. It was partially explained by serum B12 (12% variance, p < 0.001), but not by *Helicobacter*-status (gastric-atrophy uncommon in IP) or levodopa treatment. Immune-inflammatory activation increases homocysteine production. Since an estimated 60% of probands are hydrogen-breath-test positive, SIBO, with its increased bacterial utilisation of B12, is a likely cause. Thus, two prognostic indicators in established IP fit with involvement of *Helicobacter *and SIBO.

## Background

It has been proposed [1] that, whatever the aetiological insult or affected brain area, a two-phase neuroinflammatory process, containment and progression, is common to neurodegenerative diseases. These two phases may predicate on systemic inflammation.

Whilst local brain inflammation does not usually signal out [2], systemic inflammation can communicate with the brain's immune system, by evading or compromising the blood brain barrier [1,3]. Idiopathic parkinsonism (IP) is associated, in small studies, with a lower blood lymphocyte count [4-7]. Morphological and neurochemical changes, characteristic of IP, are seen in the enteric nervous system [8,9]. IP also involves heart [10] and skin [11,12], and endocrine [13,14], metabolic [15,16] and peripheral inflammatory processes [13,17]. There is a long prodrome [for review see [18]]. The 'infection hypothesis' for IP implicates the gut, the '*Helicobacter *hypothesis' one player [18]. Slow gastrointestinal-transit precedes IP-diagnosis and becomes increasingly-apparent after [19,20], predisposing to small-intestinal-bacterial-overgrowth (SIBO) [21]. The serum *Helicobacter *antibody immunoblot-profile predicts presence, severity and progression of IP [22]. Conversion of malignant IP to benign following *Helicobacter *eradication is reported, irrespective of presence of anti-parkinsonian medication [23,24]. Marked deterioration accompanied eradication-failure [23]. Attributing the benefit of *Helicobacter *eradication solely to levodopa absorption [25], ignores the effect in those not receiving this short t1/2 dopamine precursor, or, indeed, any anti-parkinsonian medication [18,23].

Confirming relative lymphopenia in probands would indicate a xenobiotic, nutritional or infective influence, finding it in their spouses a shared insult in adult-life. In IP, there is no convincing evidence of excessive exposure to xenobiotics, or of the relevance of genes regulating their metabolism [18]. There is no indication of malnutrition/malabsorption, but increased energy expenditure may cause weight loss [24]. Regarding possible haematinic deficiency, peptic ulcer is prodromal [26]. Lymphopenia might result directly from chronic infection. *H. pylori *whole-bacteria, cell-fractions, culture-supernatants and protein-products, and *H. pylori*-specific regulatory T-lymphocytes inhibit human T-cell proliferation [27,28]. Moreover, a lower serum IgM is associated with *Helicobacter*-seropositivity [29], but whether the relationship holds in IP is unknown. Lymphopenia could result from autoimmunity, reversible by eradicating a trigger, as proposed in *Helicobacter*-associated idiopathic thrombocytopenic purpura (ITP) [30]. Indeed, antibody against a *H. pylori *virulence-factor, cytotoxicity-associated-gene (*cagA*) product, is implicated in ITP [30] and IP [22]. In the presence of SIBO, most IP-probands' duodenal enterocytes contain apparently hypertrophic mitochondria [18]: lymphocyte mitochondria might show similar morphological compensation for hypofunction [31].

We present the first comprehensive overview of the blood profile in IP in relation to indices of *Helicobacter *infection and hydrogen-breath-testing for SIBO. We consider definition of a 'premonitory' stage, potential markers of progression towards established disease and prognostic indicators within it. An aetiological/pathogenic solution may remain elusive without embracing limited manifestations in 'controls'.

## Methods

### Patients and controls

In phase-1 [22], we recruited consecutive patients with 'clinically-definite' IP [32], criteria as in Table 1. There was a contemporaneous call for healthy controls without IP: those found to have 'clinically-possible or -probable' parkinsonism [32] (Table 1, footnote^a^) were not excluded. Other criteria were as shown for probands in the Table. Serum immunoglobulin concentrations were contrasted between 120 probands (12 men, 12 women per decade, 40-89 years) and 205 controls (≥12 each gender per decade, 30-89 years). The relationship of presence/absence of features of clinically-possible or -probable IP to concentration was explored in controls, as was that of a continuous measure of hypokinesia, mean-stride-length at free-waking-speed [33]. Any differential effect of *Helicobacter *anti-urease antibody-status was examined.

**Table 1:** Inclusion and exclusion criteria for probands.

Inclusion		
1.	Independently-living subjects with clinically-definite^a ^idiopathic parkinsonism	

2.	Caucasian with English as first language and living in UK^b^	

**Exclusion**		

1.	Secondary parkinsonism, "parkinsonism-plus" syndromes and other wider clinical entities [32]	

2.	Clinical depression [64], dementia [65,66], or other mental illness.	

3.	Other specific neurological condition	

4.	Exposure to specific antimicrobial/anti-secretory therapy against *Helicobacter*	

5.	Inflammatory bowel disease or history of major gastrointestinal surgery	

6.	Other progressive or resolving disorders affecting physical ability or performance^c ^or sufficient underlying incapacity to prevent assessments (e.g. use of walking aid)	

7.	Cardiovascular/respiratory symptoms during normal activities	

8.	UK MRC muscle strength score <4/5	

9.	Arthropathy, mucsulo-skeletal disorder or overt abnormalities of/history of orthopaedic surgery to joints of spine or lower limbs	

10.	Concurrent therapy with drugs which might be anti-dopaminergic or with hypnotics or sedatives	

11.	Recent change in life situation (e.g. bereavement or change in marital status/domicile)	

^a^Any combination of three of the cardinal features: resting tremor, rigidity, bradykinesia or impairment of postural reflexes. Alternatively sufficient two of the four features, with one of first three asymmetrical [32]. Responsiveness to a dopaminergic drug challenge not a requirement. Excluded were:-

• clinically-probable parkinsonism. Combination of any two cardinal features. Alternatively, asymmetrical resting tremor, rigidity, or bradykinesia sufficient.

• clinically-possible. Presence of any one: tremor, rigidity, or bradykinesia. Tremor must be of recent onset, may be postural or resting.

N.B. In controls in Phase 1, presence/absence of following features [53,67] judged (unilateral being sufficient) by two physicians:-

(i) hand bradykinesia* (obvious slowing pronation/supination).

(ii) rigidity* at rest and/or activation phenomenon (rigidity evoked by voluntary movement of contralateral limb).

(iii) rest tremor* and/or postural tremor (over a 20 m walk).

(iv) forward displacement of occiput after standing for 1 minute, with buttocks and heels against a wall (posture abnormal if distance >10 cm).

*evaluated in upper limbs, with seated subject's head, trunk and lower limbs screened.

^b^To constrain ethnic and/or geographical influences.

^c^Assessments and blood sampling avoided during acute intercurrent illness.

In phase-2 [23], we obtained first-visit full blood counts (FBC) from 118 other clinically-definite IP-probands (Table 1). These were contrasted with 381 routine FBCs from consecutive adults, requested by general practices from the same laboratory over the same period. Exclusions in these controls were limited to specified parkinsonism; haematological, immunological and malignant disorders; haemorrhage; and race other than Caucasian (see footnote^b^). FBCs from 87 accompanying spouses/partners of probands were also studied, inclusive of clinically-possible/probable/definite IP (other criteria as for probands). Proband and 'spouse' lymphocyte subset data were contrasted with summary statistics (kit manufacturer's and published [34]) for haematologically-normal adult donors. The following were explored as determinants of FBC and subset profiles: serum haematinic and homocysteine concentrations (probands); serum autoantibody screen (probands); *Helicobacter *indices and results of hydrogen-breath-testing for SIBO (probands/spouses). In a subgroup, within-proband time-trends in cell counts (yearly sampling) were contrasted between the urea-breath-test (UBT) positive for *Helicobacter *infection and the UBT-negative, any impact of *H. pylori *eradication being noted. Any modifying effect of prevalent autoantibodies on the outcome of *H. pylori *eradication therapy was explored. This involved duplicate gait assessments on two baseline occasions and at ≥ 6-weekly intervals post-therapy, any background anti-parkinsonian medication being constant, and usage of levodopa being an exclusion.

In both phases, any anti-parkinsonian medication and laxative use were recorded. Protocols had been approved by local ethics committees, written consent obtained from participants.

### Blood cellular profile

**Figure 1:**
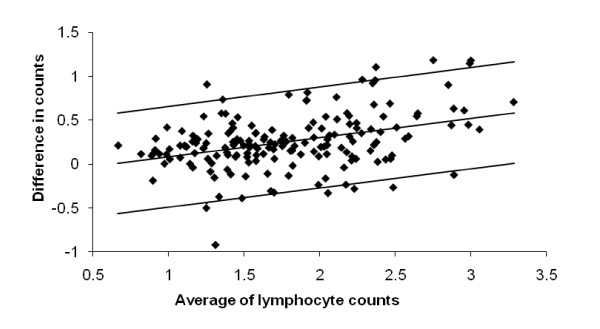
**Bland-Altman contrast of difference in and average of lymphocyte counts (×10^9^/l) by two methods in 163 subjects**. Difference expressed as subset sum (CD3+, CD19+ and CD16+56+)* minus FBC value**. Regression line and 95% upper & lower limits of agreement shown. Difference between subset sum and routine lymphocyte count is small around grand mean for average count, its variability constant over the range. *Four-colour fluorescent cell labelling using the MultiTEST kit in TruCOUNT tubes and a FACSCalibur flow cytometer (Becton Dickinson, San Jose, California, USA). ** Using XE2100, Sysmex UK Ltd., Wymbush.

Whole blood T-cell (CD3+), T-helper (CD4+), cytotoxic T-cell (CD8+), B-cell (CD19+), and natural-killer (NK) cell (CD16+CD56+) counts were determined. The sum of the lymphocyte subsets was validated by reference to FBC lymphocyte count [35] (Figure 1).

### Serum haematinics and homocysteine

Sera were stored at -20°C, and randomly allocated within-assay, with (except for *Helicobacter *serology, see below) between-assay stratification for subject-group, age and gender.

Serum ferritin was measured by two-site sandwich immunoassay with two anti-ferritin antibodies; folate, vitamin B12, and homocysteine by competitive immunoassays, using direct chemiluminescence (Bayer Diagnostics Europe Ltd, Newbery, UK.) Inter-assay coefficients of variation (CV) were 2.7, 4.2 and 3.7% at 10.8, 142.4 and 929.6 μg/l ferritin, respectively; 6.11, 7.19 and 6.36% at 1.70, 10.01 and 14.95 μg/l folate; 9.2, 2.7 and 3.0% at 178.7, 608.83 and 1343.87 ng/l B12; and 5.2, 1.5 and 3.1% at 4.9, 22.2 and 61.6 μmol/l homocysteine.

### Serum immunoglobulin classes and autoantibodies

Immunoglobulin classes were measured by immunoturbidity (Cobas Mira Plus, Roche Diagnostics Corp., Indianapolis, USA). Inter-assay CV was 6.5 and 4.9% at 7.5 and 11.6 g/l IgG, respectively; 5.9 and 4.4% at 1.3 and 2.5 g/l IgA; and 4.5 and 3.4% at 0.7 and 1.1 g/l IgM. IgA subclasses were measured by radial immunodiffusion (Bind A Rid, Birmingham, UK): CV was 11% at 3360 mg/l IgA1; and 7.5% at 360 mg/l IgA2. Serum was screened for anti-nuclear, anti-smooth muscle, anti-mitochondrial, anti-gastric parietal cell and anti-liver/kidney microsomal antibodies. The additional autoantibodies listed, as well as the above, were sought before *H. pylori *eradication therapy and on average 1 1/2 years after, in a subgroup: anti-neutrophil cytoplasmic (ANCA), anti-thyroid peroxidase, anti-adrenal, anti-tissue transglutaminase, anti-intrinsic factor, anti-acetylcholine receptor, anti-neuronal nuclear and anti-purkinje cell. For each assay, samples were processed in a single batch, blind to sequence.

### *Helicobacter *status

*Helicobacter*-status was defined by:- (i) [^13^C]urea-breath-test (INFAI Ltd., York, UK). (ii) Enzyme-linked immunosorbent assay (ELISA) of IgG-antibody directed against cell-bound *H. pylori *urease (SIA417A, Delta Biologicals, Rome, Italy: seropositive 'ELISA-value' >2.2). Inter-assay CV was 13.0, 8.0 and 6.0% at ELISA-values 0.8, 2.4 and 5.9. (iii) Western Blotting for serum antibodies against *H. pylori *antigens (RIDA *Helicobacter *Blot IgG, Quadratech Diagnostics Ltd., Epsom, UK: score >12 positive for current infection, 11-12 equivocal, <11 negative, irrespective of band-density). Test and quality-control immunoblots were read using a smooth curve, fitted through distance/molecular weight co-ordinates derived from the kit's developed-control strip. This identifies 11 bands: *cagA *product; vacuolating-toxin(Vac)A; urease-B; outer-membrane-protein; flagellin; those at 47, 33, 29, 28, 25 & 19 kDa. Magnifying strips optimized precision. *Helicobacter *serology was standardised between study phases by protocol, equipment and quality-control, using same investigator throughout. In phase 2, *H. pylori *infection was confirmed in probands by endoscopic-biopsy for histopathology, culture and molecular-microbiology [23], eradication judged by UBT and, wherever possible, repeat biopsies.

### Hydrogen-breath-test for small-intestinal-bacterial-overgrowth

Hydrogen-breath-tests were carried out in UBT-negative probands and spouses, using a 25 g lactulose test-dose, following 24-hour deprivation of dairy products (and medicinal lactulose) and a breakfast of 250 ml black tea/coffee or water. Hydrogen concentrations were measured (Micro Medical Ltd., Rochester, UK) pre-dose and at 15 minute-intervals for 4 hours after. A prolonged test, with non-absorbable substrate, was used because of potentially impaired gastric-empting and intestinal-transit [9,24]. Breath-hydrogen/time curves [36,37] were not bimodally distributed between-subject. Within-subject, two distinct peaks, early and 'colonic', were not present. Summary outcomes were examined: 2 hour value; maximum-value between 15 and 120 minutes; linear increase over 2 hours, when trend was to peak. Correlation coefficients were high for all contrasts, the maximum-value selected for analysis.

### Statistical analysis

'Contemporaneous' reference range limits, constructed from a measurement made in 400 controls, would have a small standard deviation (approximately 0.09s, where s is standard deviation in the measurement), and be only marginally larger (0.1s) using 200 measurements [38].

**Figure 2:**
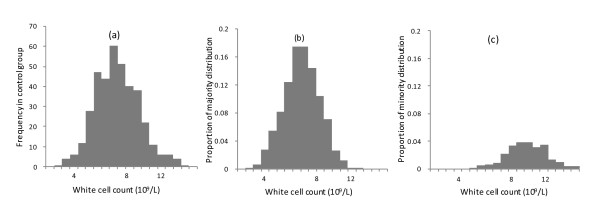
**Distribution of total white cell count, and its decomposition into two Gaussian distributions**. Histogram (a) shows log_e _transformed raw data. Histograms (b) and (c) are generated from parameter estimates obtained by applying a Gaussian mixture model to log_e _(white cell count). They represent the 'best' mixture to replicate the overall distribution: i.e. two distributions tended to provided a better fit (likelihood ratio test, 3 degrees of freedom, χ^2 ^= 7.25, p = 0.06), (b) representing 80% of data, (c) a shift-to-the-right. There was no evidence that a mixture of Gaussian distributions provided a better fit to distribution of log_e _transformed lymphocyte (age-corrected) or neutrophil counts (χ^2 ^= 0.74 & 3.49, p = 0.9 & 0.3, respectively).

In phase-2, controls below age 46 years had anomalous age-trends in FBC indices: these (19, presumably relatively-ill, individuals) were excluded. Since phase-2 controls were not subject to the rigorous inclusion/exclusion criteria of phase-1, their white cell counts were examined for evidence of distinct Gaussian distributions (e.g. a subgroup with a higher mean count, compatible with acute intercurrent infection). There was a tendency for two distributions to describe total white cell count better (Figure 2), but no evidence that a mixture of distributions provided a better fit for lymphocytes or neutrophils. Comparisons with a 'conventional' reference range allowed robustness of any shift in distribution between subject-groups to be tested. Wilcoxon signed-rank test was used to assess a shift from the reference median, chi-square goodness-of-fit to test whether the expected 5% of measurements fell outside the range.

Many chronic diseases are multi-step and multi-factorial: explanatory models were used to examine between-group and within-subject differences in laboratory measurements. Potential confounding or effect-modifying variables were assessed, examining for interactions prior to direct effects.

Where indicated, logarithmic transformations were employed, to ensure validity of assumptions of normality of distribution and equality between groups of residual variance. The regression parameter for a log transformed outcome-variable was exponentiated, giving a relative (percentage) rather than absolute difference. That for a log transformed predictor was scaled by 1/log_e_2, so that a unit change reflected a doubling.

## Results

### Full blood count

**Figure 3:**
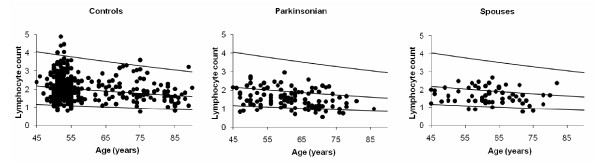
**Contrast of lymphocyte counts (×10^9^/l) in probands with idiopathic parkinsonism and their spouses with an age-specific 95% reference range based on routine requests**. Counts are corrected as if all subjects were male, using the relationship to gender in controls, there being no evidence of an age.gender interaction.

Table 2 shows that the major differences in FBC of IP-probands (P) and their spouses (Ps) from controls (C) were in total lymphocyte count. Mean count was lower by 23.8 (95% CI: 18.7, 28.7)% in P and 17.3 (10.0, 24.0)% in Ps (p < 0.001 in each case, age & gender adjusted). Figure 3 shows the estimated proportion falling below the age-specific contemporaneous reference range: this was 12.5 (binomial exact 95% CI: 7.2, 19.8)% of P and 7.1 (2.4, 15.9)% of Ps, compared with the 2.5% expected. No proband or spouse exceeded the range. The proportion falling below the lower limit of the conventional reference range (1.3 × 10^9^/l) was 30.8 (22.7, 39.9)% of P, 10.0 (4.1, 19.5)% of Ps and 7.3 (4.9, 10.4)% of C, the frequency of 'lymphopenia' varying between groups (Pearson χ^2^, p < 0.001). In the 37.0% of probands never exposed to anti-parkinsonian medication, mean lymphocyte count was 101.0 (95% CI: 89.5, 113.9)% of that in Ps, still significantly lower than in C. Exposure was associated with a 11.1 (0.1, 20.8)% lower count (p = 0.048), but inclusion of time-from-diagnosis (not important independently) reduced its significance to 0.1. Thus, medication status and time-from-diagnosis were intrinsically linked, possibly surrogate for a more pertinent measure of disease.

**Table 2:** Mean (95% data interval) for full blood count indices in probands with idiopathic parkinsonism, their spouses and contemporaneous routine requests from primary care.

	Probands**	Spouses***	Controls
	
Characteristic*	(n = 126)	(n = 79)	(n = 381)
Age (years)	60.8 (41.0, 80.5)	59.4 (37.2, 81.6)	58.4 (37.6, 79.1)

Gender	75 M: 51 F	23 M:56 F	197 M:184 F

Haemoglobin (g/dl) male	14.2 (12.1, 16.3)	14.2 (13.1, 15.3)	14.4 (11.7, 17.0)

Haemoglobin (g/dl) female	13.1 (11.4, 14.8)	13.2 (11.7, 14.8)	13.1 (11.0, 15.2)

RBC (10^12^/l) male	4.72 (3.96, 5.48)	4.70 (4.23, 5.16)	4.63 (3.72, 5.54)

RBC (10^12^/l) female	4.36 (3.72, 5.00)	4.44 (3.88, 5.00)	4.39 (3.64, 5.14)

PCV male	0.43 (0.38, 0.48)	0.43 (0.4, 0.45)	0.42 (0.35, 0.49)

PCV female	0.40 (0.34, 0.45)	0.40 (0.36, 0.45)	0.39 (0.34, 0.45)

MCV (fl)	91.35 (84.04, 98.66)	90.99 (83.24, 98.74)	90.91 (80.94, 100.87)

MCH (pg)	30.12 (27.22, 33.02)	30.03 (27.17, 32.88)	30.49 (26.41, 34.57)

MCHC (g/dl)	32.97 (31.03, 34.92)	33.00 (31.47, 34.53)	33.55 (31.50, 35.59)

Platelets (10^9^/l)	240 (151, 382)	244 (154, 386)	242 (142, 412)

White cell count (10^9^/l)	6.31 (3.79, 10.51)	6.29 (3.58,11.04)	6.66 (3.86,11.50)

Neutrophils (10^9^/l)	3.95 (1.93, 8.08)	3.78 (1.90, 7.53)	3.69 (1.77, 7.66)

Lymphocytes (10^9^/l)	1.52 (0.78, 2.97)	1.71 (0.93, 3.12)	2.05 (1.09, 3.88)

* Abbreviations specified in text.

** Presenting cases plus 8 spouses found to have clinically-definite IP. The latter tended (p = 0.07) to have a higher lymphocyte count.

*** Remaining spouses.

Neutrophil polymorphonuclear count tended to be higher in P (p = 0.1, gender adjusted) than in C. Haemoglobin concentration, red blood corpuscle count (RBC), packed cell volume (PCV), mean corpuscular volume (MCV), platelet count and mean platelet volume were not different in P or Ps from C. Mean corpuscular haemoglobin concentration (MCHC) was less in both than in C, by small but highly significant amounts (0.46 (0.27, 0.65); 0.33 (0.09, 0.58) g/dl, respectively, p < 0.001 & = 0.008, age and gender adjusted). Mean corpuscular haemoglobin (MCH) tended to be less in P (p = 0.06).

### Lymphocyte subsets

**Figure 4:**
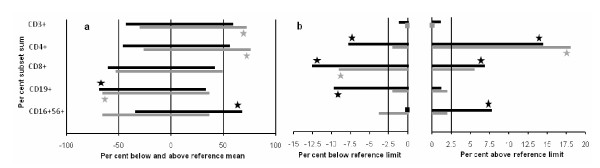
**Shifts in percentage distribution of lymphocyte subsets in probands (P), with idiopathic parkinsonism, and their spouses (Ps) compared with reference range (a) mean and (b) its lower and upper reference limits**. In P (black bar) and Ps (grey bar), significant differences are denoted by black star and grey star, no subject outside reference limit by black square and grey square. In (b), more than the 2.5% expected had CD4+ (in P), CD8+ (P & Ps) and CD19+ (P) below lower limit (exact binomial test: p ≤ 0.01 in each case), and CD4+ (P & Ps), CD8+ (P) and CD16+56+ (P) above upper limit (p ≤ 0.02).

Subset counts in 106 of P and 70 Ps, expressed as a percentage of subset sum, are compared with the kit manufacturer's reference mean and 95% confidence limits in Table 3 and Table 4. Figure 4 summarises direction of effects. In P, CD4+ and CD8+ distributions were platykurtic, greater proportions than expected falling both above and below reference ranges. In Ps, there was a significant rightward shift in mean CD4+, and a significant leftward shift in CD8+ with respect to lower reference limit. A significant leftward shift in mean CD19+ was seen in both P and Ps. That to-the-right in mean CD16+56+ characterised P alone. Subset shifts were not explained by anti-parkinsonian medication.

**Table 3:** Comparison of percentage distribution of lymphocyte subsets in probands with idiopathic parkinsonism and their spouses with Multitest reference mean.

Subset	Reference mean	Probands		Contrast with reference	Spouses		Contrast with reference
	
	% subset sum	% below	% above	p-value*	% below	% above	p-value
CD3+	72	42.5	57.5	0.5	28.1	71.9	0.0007

CD4+	45	45.3	54.7	0.09	24.6	75.4	0.0001

CD8+	24	59.4	40.6	0.06	52.6	47.4	0.3

CD19+	13	67.9	32.1	0.0001	64.9	35.1	0.003

CD 16+56+	14	33.0	67.0	0.0001	63.2	36.8	0.2

* Wilcoxon signed-rank test assuming reference mean ≡ median.

For example: in probands, CD3+ subset conformed with reference mean value of 72% and range (see Table 4) of 56-86%, in their spouses with range but not mean.

**Table 4:** Comparison of percentage distribution of lymphocyte subsets in probands with idiopathic parkinsonism and their spouses with Multitest reference range.

Subset	Reference range	Probands			Goodness of fit	Spouses			Goodness of fit
	
	% subset sum	% below	% within	% above	p-value*	% below	% within	% above	p-value
CD3+	56-86	0.95	98.1	0.95	0.3	0.0	100.0	0.0	0.2

CD4+	33-58	7.6	78.3	14.1	0.0001	1.8	80.7	17.5	0.0001

CD8+	13-39	12.3	81.1	6.6	0.0001	8.8	86.0	5.2	0.004

CD19+	5-22	9.4	89.6	1.0	0.0001	1.7	96.5	1.8	0.9

CD 16+56+	5-26	0.0	91.5	8.5	0.0001	3.5	94.7	1.8	0.8

*Pearson's χ^2^

No difference in CD4+/CD8+ ratio was found between P and Ps, after adjustment for higher values with age (p = 0.001) and lower values in males (p = 0.004). Overall, ratios complied with the inter-quartile reference range for gender by decade [34]. However, in younger males (41-50 years; 14P, 2Ps), the mean ratio (1.26 (0.92, 1.61)) was markedly below the expected 2.29 (p < 0.0001). In the 14 younger male probands, the lower the ratio, the higher the CD8+ count (adjusted *r*^2 ^= 40%, p = 0.01), but not the lower the CD4+.

Regarding within-couple concordance (70 pairs), CD3+, CD4+, CD8+ and CD19+ counts were lower in probands, by 14.2 (2.0, 24.9), 18.2 (5.1, 29.5), 17.1 (2.1, 29.8) and 31.9 (14.4, 45.8)% respectively (paired t-tests: p = 0.03, 0.01, 0.03 & 0.002). Probands' CD16+56+ count was higher by 23.6 (7.7, 42.0)% (p = 0.004).

### Duodenal and lymphocyte mitochondria

**Figure 5:**
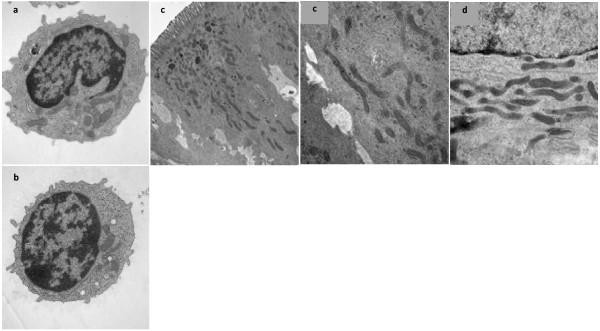
**Lymphocyte and duodenal enterocyte ultrastructure in idiopathic parkinsonism (IP)**. Electron micrographs shows mitochondria in a representative blood lymphocyte from (a) a man with clinically-definite lP and (b) an age-matched healthy man, and in a representative duodenal enterocyte from (c) this IP-proband (panel to right shows higher magnification) and (d) his spouse. The proband's normal lymphocyte mitochondria are in marked contrast to his and his spouse's long thin duodenal mitochondria. The proband's IP had been diagnosed 7 years previously. He was receiving anti-parkinsonian medication. His spouse had probable-IP on screening. Both had bloating and cyclical diarrhoea going back 10 years. Typically, in the proband, 4 days of unformed stool alternated with constipation in a four-week cycle. In the spouse, explosive watery diarrhoea followed abdominal cramps, over 2 days in 2 week cycles, with normal bowel habit in between. Both had a positive hydrogen-breath-test (criterion: two consecutive values [37] >cut-point of meter manufacturer), and were negative for *Helicobacter *by UBT, serology, and culture/molecular microbiology on gastric biopsy.

Figure 5 illustrates that the long thin duodenal enterocyte mitochondria found in IP are not necessarily associated with abnormal lymphocyte mitochondrial morphology.

### Serum haematinic and homocysteine concentrations

Mean nutrient intakes were estimated from 3-day food diaries in 23 of the phase-2 probands: they complied with UK National Reference Values.

**Figure 6:**
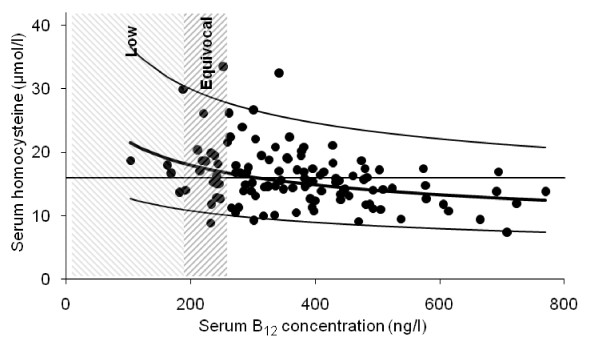
**Relationship of serum homocysteine to B12 concentration in idiopathic parkinsonism**. Two probands with exceptionally high homocysteine (110 and 135 μmol/l) are excluded: the first had a low B12 but normal folate, the second (with frank *H. pylori *infection and an empyema) a low folate with a normal B12. Neither had received levodopa.

In all 126 probands (Table 2), serum homocysteine was above the desired-maximum of <16 μmol/l in 43.2 (binomial exact 95% CI: 34.1, 52.7)%. Figure 6 shows that the expected proportion had a serum B12 below reference range (<180 ng/l), but 16.1 (10.0, 24.0)% had values within the 'equivocal range' (180-250 ng/l). The variance in homocysteine explained by B12 was only 12.1% (adjusted *r*^2^, p < 0.001). Including relevant covariates, time-from-diagnosis (p = 0.001) and gender (p = 0.01) in the model increased variance explained to 27.0%, B12 maintaining its significance. Time-from-diagnosis was independent of age, which itself did not contribute to variance explained. Homocysteine was 1.8 (95% CI: 0.8, 2.8)% higher per year post-diagnosis, 12.5 (2.5, 22.5)% higher in females.

Six percent of probands were both newly diagnosed and untreated: their median homocysteine concentration, 13.4 μmol/l, was below the desired maximum. Receiving anti-parkinsonian medication other than levodopa (40.3%) was not associated with increased homocysteine, compared with being untreated (37.0%). Probands receiving levodopa (22.7%) had a median total daily dose of 300 (interquartile range 300, 450) mg), a mean time-from-diagnosis of 7 years. Their homocysteine was higher than in the remainder by 24.2 (95% CI: 9.6, 40.8)% (p = 0.001, adjusted for B12 & gender). However, exposure to levodopa was intrinsically linked to time-from-diagnosis, and the estimated 7-year increase in homocysteine for probands never exposed to levodopa (19.6 (7.7, 31.6)%) was comparable.

Probands' serum folate distribution was platykurtic, 7.6 (binomial exact 95% CI: 3.5, 14.0)% having concentrations above the reference range (3-13 μg/l), 13.6 (8.0, 21.1)% below. Variance in homocysteine explained by folate (p = 0.007) increased from 6.1 to 21.0% by including time-from-diagnosis (p < 0.001) and gender (p = 0.005) in the model, but the significance of folate decreased to 0.1. Folate did not contribute to variance explained by B12, gender and time-from-diagnosis.

Serum ferritin was below the reference range (20-300 μg/l males; 20-200 μg/l post-menopausal females) in 6.8 (3.0, 12.9)%. It contributed to explaining probands' MCHC and MCH: estimated increase of 0.17 (95% CI: 0.01, 0.33) g/dl in MCHC and 0.30 (0.06, 0.54) pg in MCH with a doubling of ferritin (p = 0.04 & 0.02, respectively).

Neither homocysteine nor these haematinics contributed to explaining total lymphocyte count in IP.

### Prevalence of autoantibodies

Anti-nuclear antibody (ANA) was present in 25.3% of the 126 phase-2 probands, anti-intrinsic factor in 15.1%, and anti-gastric parietal cell in 7.6%. Gastric autoantibodies were not associated with a lower serum B12. Anti-smooth muscle antibody was present in 2.6%, anti-mitochondrial and anti-liver/kidney microsomal negative throughout. In the more comprehensive screen in 21 probands, ANCA was present in 3, but none had antibodies against myeloperoxidase or proteinase-3 targets. Total lymphocyte count was not associated with autoantibody-status.

### Serum immunoglobulin class concentrations

Table 5 shows that, in phase-1, serum immunoglobulin concentrations in the 120 IP-probands and 205 contemporaneous controls were similar.

**Table 5:** Serum immunoglobulin concentrations in probands with idiopathic parkinsonism and controls.

Serum concentration	Geometric mean (95% data interval)^a^		
		
	Probands	Controls	Covariates^b^
		
	n = 120	n = 205	
IgM (g/l)	0.92 (0.30, 2.79)	0.90 (0.24, 3.35)	age, *Helicobacter *status*

IgG (g/l)	11.39 (6.11, 21.21)	11.92 (7.33, 19.39)	gender**

IgA (g/l)	2.95 (1.12, 7.79)	3.13 (1.15, 8.46)	age, gender***

IgA1 (mg/l)	1574 (538, 4606)	1652 (569, 4797)	

IgA2 (mg/l)	406 (106, 1557)	388 (97, 1552)	

Demographic definition:- Mean age: 71 (sd 11.7, range 40-89) years in probands, 65.0 (15.9, 30-89) in controls. Percentage of males same (51%). Height not significantly different. Weight greater in controls (mean difference 4.00 (95% CI: 1.25, 6.75) kg). Affect score (20-point: median 5 (interquartile range 9, 3) in probands & 3 (5, 2) in controls) and cognitive function score (16-point: 14.5 (12.8, 15.5) & 15 (14.5, 16)) were consistent with exclusion of depression and clinical dementia. Median (interquartile) social status, by UK Registrar General's Social Classification, was the same in probands and controls. Severity of parkinsonism, by Hoehn and Yahr [40] Staging (I-V) of functional impairment: 31% of probands stage II, 38% stage III, 31% IV. Individual anti-parkinsonian drug categories used were levodopa/decarboxylase inhibitor combination (81.7% probands), dopamine agonist (20%), selegiline (55%) and antimuscarinics (17.5%), whilst 7.5% were untreated.

^a ^Values adjusted to age 60 years, and as if all subjects male.

^b ^Previously determined in controls [29]:-

* IgM decreased overall by 1.04 (95% CI: 0.50, 1.58)% per year (p < 0.001). IgM was lower by 19.75 (3.80, 33.05)% (p = 0.02) in *H. pylori *ELISA-seropositive than in seronegative. Effect of seropositivity equivalent to 25 years of ageing, irrespective of current age.

** IgG higher in males (p = 0.002).

*** IgA higher in males (p < 0.001), and increased with age (p = 0.007). Relationship of IgA1 to age was quadratic (p = 0.003, with respect to quadratic term), decreasing towards an estimated turning point of 57 years, then increasing. Relationship of IgA2 to age had quadratic trend (p = 0.1), in mirror image with similar turning point.

**Figure 7:**
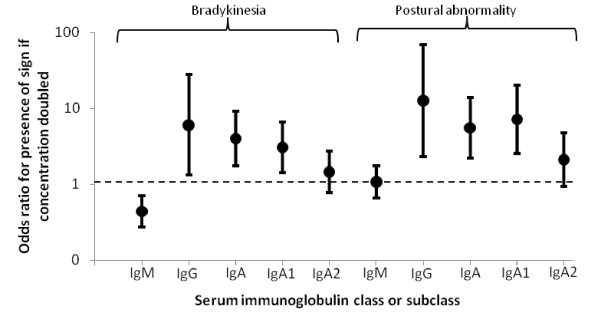
**Estimated odds ratio, with 95% CI, in subjects without clinically-definite parkinsonism, for presence of a given sign, if serum immunoglobulin concentration doubled**. Concentrations adjusted to age 60 years and as if all subjects male. Statistical significance of associations with bradykinesia and postural abnormality are, for IgM p = 0.001 & 0.8; for IgG 0.02 & 0.003; for IgA 0.001 & 0.001; for IgA1 0.004 & 0.001; for IgA2 0.2 & 0.07, respectively. Regarding rigidity, presence of activation phenomenon associated with IgA (OR = 1.7 (1.1, 2.6), p = 0.01) and IgA1 (1.6 (1.1, 2.3), p = 0.02).

Figure 7 shows that presence, in controls, of hand-bradykinesia (16%), or postural abnormality (9%), was very strongly associated with a higher IgG and IgA. Stride-length was shorter by 59.3 (95% CI: 1.9, 116.8) mm if IgG concentration doubled (p = 0.04), but gait was not associated with IgA. Resting rigidity in arms was rare (1%), but rigidity evoked by movement of the contralateral limb, the activation phenomenon, common (70%) and associated with IgA (Figure 7 footnote). Resting tremor was also rare (1.5%), postural tremor common (48%) but not associated with immunoglobulin classes. Of these features, only hand-bradykinesia was associated with IgM, by contrast a strong negative relationship. All associations with IgA were attributable to IgA1.

Immunoglobulin concentrations, in probands, were unrelated to the presence/absence of a cardinal feature or stride-length. Concentrations were not associated with any class of dopaminergic medication. However, constipation, as denoted by regular laxative use (22 of the 100 probands so questioned), was associated with increased IgA (odds ratio 2.1 (1.03, 4.4) for concentration being double if taking medication, p = 0.04). Antimuscarinics can, of course, constipate: the odds ratio remained similar (2.30 (1.23, 4.33), p = 0.01) when antimuscarinic use (14 more probands) was also taken to denote constipation. Again, relationships depended on IgA1.

### *Helicobacter*-status of spouses and probands

Crude prevalence rate estimates for 'Parkinson's disease' in 34 European studies range widely, from 65.6 to 12,500 per 100,000 [39]. The estimate lay within the binomial exact 95% CI for the spouse cohort (9,195 (4,045, 17,317) per 100,000) in only one study. All 8 spouses with clinically-definite IP had 'frank' UBT-positive *Helicobacter *infection, only 8% of the other spouses did (*P *< 0.0001). Table 6 shows that frequency of positive *Helicobacter*-status, by breath-test or serology, tended to be less in the spouse cohort than in presenting cases.

**Table 6:** Comparison of prevalences of *Helicobacter*-positivity, by urea-breath-test (UBT) and serology, between probands with idiopathic parkinsonism and their spouses and controls.

	*Helicobacter*-positivity (%)					
Test	Probands		Spouses		Controls	
	Phase-1†	Phase-2††	Phase-1	Phase-2††	Phase-1†	Phase-2
	**n = 120**	**n = 118**		**n = 86^a^**	**n = 196^b^**	

UBT	-	29.6	-	16.9	-	-
ELISA	47.6	34.5	-	18.4	39.5	-
Immunoblot^c^	36.7	50.0	-	34.2	30.8	-

^a^including 8 with clinically-definite IP.

^b^without clinically-definite IP.

^c^equivocal score in a further 8.2% of Phase 2 subjects.

† prevalence of seropositivity not significantly different. Both ELISA and immunoblot available in 196 of 205 controls.

†† p = 0.1, 0.06 & 0.06 (age & gender adjusted) for comparison of prevalence of positive-status by UBT, ELISA & immunoblot, respectively.

N.B. Absolute change in prevalence of *Helicobacter *not addressed since IP-cohorts not matched for locality/social class.

In the earlier IP-cohort, prevalence of seropositivity was not significantly different from that in the socially-similar controls (Table 6). The odds of a proband's sample being immunoblot-positive and ELISA-negative, compared with *vice versa*, became 69.1(95% CI: 4.9, 978.3) times greater in phase-2 than, 10 year earlier, in phase-1 (p = 0.02). In the later IP-cohort, ELISA-positivity was shown to represented frank infection, in that its prevalence concurred with that of UBT-positivity.

### *Helicobacter *and blood profile

#### Cellular profile

In the later IP-cohort and their spouses (Table 6), UBT-positivity was not associated with total lymphocyte count (nor was ELISA-positivity). However, the CD8+ subset count was higher with frank infection by 27.8 (6.1, 53.9)%, (p = 0.01, age adjusted: no interaction between presence/absence clinically-definite IP and *Helicobacter*-status, nor any direct disease-status effect). Immunoblot-positivity was much more frequent and associated with a higher total lymphocyte count (by 11.9 (2.2, 21.7)%, p = 0.02, age & gender adjusted). Even an equivocal immunoblot score tended to be (16.3 (-1.4, 33.9)%, p = 0.07). Of the 11 bands sought, only anti-VacA (39.4%) was relevant to total count (p = 0.01). A positive/equivocal immunoblot was associated with a higher CD3+ count (p = 0.03). It was associated with a higher CD19+ count only in the presence of frank infection (immunoblot-status.UBT-status interaction, p = 0.01). The CD16+56+ count was not associated with *Helicobacter*-status.

With UBT-positivity, platelet count was higher (9.2 (1.2, 17.8)%, p = 0.02, gender adjusted) and serum folate lower (25.2 (7.9, 39.2)%, p = 0.007: measured in probands only). This is compatible with bleeding and gastritis. However, MCHC and serum ferritin were not associated with *Helicobacter*-status, neither were B12 and homocysteine.

Serial lymphocyte counts were obtained in 49 probands (203 observations, median 4 (interquartile range 3, 5), over 2.6 (2.1, 3.5) years), counts were stable in the 21 without frank infection. However, following biopsy-proven *H. pylori *eradication in 28, lymphocyte count did tend to increase (3.6 (95% CI: 0.0, 7.8)% per year, p = 0.08), there being no prior time-trend.

#### Autoantibody-status

Neither UBT- nor immunoblot-status influenced the frequency of ANA, the most common autoantibody found in clinically-definite IP.

Of 21 biopsy-positive probands, screened for autoantibodies in relation of anti-*H. pylori *therapy, ANA was present in 8 before, 10 after. Eradication failed in 6 (2 UBT-positive, 4 biopsy-positive only), of whom 5 were ANA-positive afterwards: ANA tended to mark continuing infection (p = 0.06). Change in mean stride-length following therapy was measured in 20 of 21, over a median of 358 (interquartile range 148, 498) days with 11 (8, 20) paired assessments. Stride-length deteriorated markedly in the ANA-positive (by 149 (95% CI: 13, 284) mm/year), in contrast (p = 0.03) to improvement in the ANA-negative (77 (-1, 156) mm/year). Anti-nuclear antibody appeared to mark a poor prognosis, but effects of ANA-status and success/failure of eradication on stride-length were intrinsically linked. A lower CD8+ or double-positive CD4+CD8+ count was also associated with deterioration in stride-length (p = 0.02, 0.03 respectively, age/gender adjusted as appropriate), and tended to be with eradication failure (p = 0.06, 0.04). Indeed, a lower CD8+ count appeared linked with ANA-positivity (p = 0.07). Neither CD16+56+ count nor serum homocysteine were prognostic indicators in this context.

#### Immunoglobulin class concentrations

Serum IgM concentration was lower with ELISA-positivity in phase-1 controls (Table 5, footnote), but not in clinically-definite IP (p = 0.02, for subject group.*Helicobacter*-status interaction). This interaction also obtained when *Helicobacter*-status was defined by the immunoblot, in particular by the presence/absence of a single (47 kDa) band (p = 0.008). Notably, the same band explained the immunoblot association with IgM in controls (p = 0.001). There was no evidence that other infections, consequent on functional impairment, were responsible: IgM was not higher the greater the Hoehn and Yahr Staging of IP [40]. No effect of subject group on other immunoglobulin classes was found, relative to *Helicobacter *serology or directly.

In controls, *Helicobacter *serology explained neither the relationship of features of clinically-possible/probable IP to immunoglobulin concentration (Figure 7) nor their presence directly.

### Small-intestinal bacterial-overgrowth and blood profile

Maximum breath-hydrogen between 15 and 120 minutes (median 41 (interquartile range 17-73) ppm) was not influenced by age, gender or subject group (40 probands, 15 spouses without clinically-definite IP, in phase-2). The meter manufacturer's diagnostic cut-point (20 ppm increment) was exceeded at least once within the 2 hours in 65% (irrespective of subject-group), by two consecutive readings [37] in 60%.

Although the maximum was not associated with total lymphocyte count, it was with the CD4+ subset count (estimated increase 9.2 (95% CI: 1.2, 17.8)% with a doubling of breath-hydrogen, p = 0.03, gender-adjusted, irrespective of subject group). It contributed to explaining probands' serum ferritin, and MCHC in probands and spouses (estimated decreases of 20.2 (8.5, 30.4)% in ferritin and 0.20 (0.02, 0.39) g/dl in MCHC with a doubling of breath-hydrogen, p = 0.03 & 0.02, respectively). Probands' folate, B12, homocysteine and autoantibody-status were unrelated to maximum breath-hydrogen. Probands' and spouses' CD4+/CD8+ ratios were not explained by breath-hydrogen (nor UBT-status or immunoblot-status).

*Helicobacter *infection appeared to keep SIBO at bay. Breath-hydrogen was higher (Spearman rank correlation, p = 0.003) in those of the currently UBT-negative probands and spouses who had been UBT-positive on presentation. It tended to be higher (p = 0.06) where the immunoblot score had been positive/equivocal. Hydrogen-breath-tests were performed, in two subsequent probands, before and serially after biopsy-proven eradication of *Helicobacter *(initially detected by molecular-microbiology on two culture-negative biopsies). One had become positive (two consecutive values criterion) at 8 months, the other converted between 12 and 18 months: even low-density infection appeared protective.

## Discussion

Table 7 summarises individual clues yielded from the blood profiling with reference to a 'premonitory' stage, and prognostic indicators within established IP. Corresponding roles for pathogens in the evolution are postulated on the basis of the background, results and discussion below.

**Table 7:** Summary of clues from blood profile to role of infection in aetiology/pathogenesis of idiopathic parkinsonism.

Blood element	Clue	Postulate
Blood lymphocyte count	Reduced count in probands and their spouses *cf *contemporaneous controls.	
		Viral origin would fit, especially in context of:- (i) prevalent abnormal bowel function, starting prodromally in probands, and found in spouses (ii) evidence of premonitory parkinsonian state in spouses.
Serum Immunoglobulin (IgM, IgG, IgA) concentrations	Strong associations with sporadic cardinal features of parkinsonism in controls suggest premonitory infection.	

Serum IgM concentration	Differential effect of *Helicobacter *seropositivity between probands and controls.	Sequestration to site gastric inflammation no longer obtains in established parkinsonism or there is increased production of poly-specific IgM (in response to *Helicobacter *or SIBO).

Serum autoantibody titres	ANA associated with failure of, and functional deterioration after, *Helicobacter *eradication therapy in probands.	Autoimmune element to response to *Helicobacter *in probands. Hence, importance of residual low-density infection.

Serum haematinic and homocysteine concentrations	Elevated homocysteine prevalent in probands: explained only in small part by haematinics.	Immunoinflammatory activation likely cause. Importance of SIBO suggested by association of breath-hydrogen with iron absorption (ferritin & MCHC markers) in a setting where moderate/severe gastric atrophy uncommon. Reduction of gastric acid by inflammatory cytokine likely mechanism.

Reduced total lymphocyte count in clinically-definite IP-probands (even when never exposed to anti-parkinsonian medication) and their spouses is a robust finding. Suppression of T-cell proliferation by *Helicobacter *[27,28] does not appear responsible. There was no cross-sectional association between lymphocyte counts and *Helicobacter*-status, although probands' total count tended to increase following eradication. No relationship was found between total count and hydrogen-breath-test result, but the impact of SIBO eradication/reacquisition was not investigated. Generalised autoantibody seropositivity would have supported an autoimmune pathogenesis for IP: its absence cannot exclude. Whilst dysfunction of lymphocyte mitochondria is reported in IP [31], they do not necessarily exhibit the abnormal morphology seen in enterocytes. Haematinics, or immuno-inflammatory activation as flagged by homocysteine [41], did not account for the reduced total count.

Unexplained reduction in lymphocyte count and prodromal enteric-nervous-system damage [19,20] might be viral in origin. Whereas ascent via gastrointestinal neural pathways [8] would fit with 'cold neurodegeneration', dissemination in blood could explain constitutional symptoms and widespread pathology. B-cells could be a target (as with Epstein-Barr virus): their proportion was reduced in spouses, more so in probands. Natural-killer cells provide first-line viral defence [42]. Probands had an increased proportion, whereas spouses had an increase in CD4+. Given the same insult, this suggests a difference in immune competence/regulation. Cytotoxic T-cells have an important role in eliminating virus-infected cells. Their platykurtic proportional distribution in probands may encompass an increase in earlier stages (as in asymptomatic human immunodeficiency virus, HIV, infection) and suppression in later stages (as in acquired immunodeficiency syndrome, AIDS). A low CD4+/CD8+ ratio can flag viral infection, classically HIV [34], and has been reported in IP [4,5]. Here, addressing age/gender covariates, it was low only in younger male probands and spouses.

We have classified intracellular microbial targets in IP for current diagnostic assays [18]. These include enteroviruses, which infect via the gastrointestinal tract and can have neurological consequences. In post-poliomyelitis syndrome, there is systemic illness with raised serum inflammatory markers, and metabolic and skin involvement [43-45]. Parkinsonism is reported [46], lymphopenia not. The century-old proposal linking poliomyelitis with motor-neurone disease has been updated to encompass subclinical infection [47]. Increasing mortality from motor-neurone disease, where vaccination has triumphed over clinical poliomyelitis [48], does not preclude this. Searching for undiscovered viruses requires leads. The epidemiology of IP and HIV are distinct, but parkinsonism is seen in uncomplicated HIV-infection (not just with opportunistic infections in AIDS), and jejunal autonomic denervation described [18]. Thus a relatively benign retrovirus might explain the lymphocyte profile and predisposition to SIBO in IP-probands and spouses.

The relative lymphopenia in spouses complements our finding of marked differences (physiological/psycho-motor/dermatological) relevant to parkinsonism between spouses, cohabiting with probands for half-a-century, and control couples [12,33,49,50]. Those spouses were, on average, a short, albeit highly significant, 'distance-down-the-pathway'. Their multifarious manifestations are difficult to dismiss as selective mating or learned/reactive behaviour. Here, both probands and spouses had a high frequency of hydrogen-breath-test-positivity. Indeed, we have estimated that 53% of probands and 36% of spouses have chronic bowel dysfunction by stringent definition [51]. Seven percent of probands, 14% of spouses have diarrhoea (unformed stool during ≥3/4 past year, plus ≥3 bowel movements/day for 1/2). Greater enteric (± vagal nucleus) neuronal damage in probands may stave-off a diarrhoeal response to SIBO. The implication is of a 'premonitory' stage in spouses, alone lacking impetus to reach the diagnostic threshold. Lack of within-couple concordance in *Helicobacter*-status between probands and spouses [23] suggests that any 'conjugally-transmitted primer' is not *Helicobacter*. However, transmission of a 'primer' to a *Helicobacter*-colonised recipient may result in progression to the diagnostic threshold. Both might be necessary, neither sufficient. Indeed, here, spouses had a relatively high prevalence of clinically-definite IP, and all so affected had frank *Helicobacter *infection. Persistence and burden of *Helicobacter *in established IP are discussed below.

Strong associations of sporadic cardinal features of parkinsonism with changes in circulating immunoglobulins also indicate a premonitory active process. That IgA associations were accounted for by IgA1 may indicate a bone marrow response, but does not exclude a mucosal [52]. The apparent link of constipation with IgA1 in probands suggests the latter. Frequency of defaecation begins to deviate from that of controls three decades before the median age of neurological diagnosis [20]. Moreover, a higher IgA1 was associated with activation of rigidity, a common sign in our controls, which enables "one to detect Parkinson's disease in its earliest phase" [53]. *Helicobacter *serology did not explain immunoglobulin associations, a virus and/or SIBO might. We describe a differential IgM response to *Helicobacter *between controls and probands. In IP, sequestration to the site of inflammation may no longer obtain, or there be increased production of poly-specific IgM by naïve-B- or B1-cells, despite the lower overall proportion of B-cells.

Hyperhomocysteinemia is associated with cardiovascular risk and conditions which overlap with parkinsonism: dementing illnesses [54] and depression [55]. Moderate elevation of homocysteine in nearly half of the IP-probands was explained only in small part by serum B12, with no complementary effect of folate. Hyperhomocysteinemia was unrelated to *Helicobacter*-status: absence of even moderate atrophy characterizes *Helicobacter*-associated gastritis in IP. [23,24]. The underlying cause is likely to be SIBO, in which there is increased bacterial utilisation of B12. We propose that consequent immuno-inflammatory activation drives homocysteine production [41]. Activation may increase demand for B12, such that a concentration in the 'equivocal' range should be regarded as pathological. An inflammatory response to SIBO would fit with the inverse association between breath-hydrogen and indicators of iron-absorption. Interleukin-1β, for example, acts centrally, as well as on gastric receptors, to inhibit acid secretion and hence iron-absorption [56]. Indeed, exploratory studies suggest a greater prevalence in IP of cytokine-gene polymorphisms predisposing to an intense innate inflammatory response [57]. Suppression of inflammation can reduce homocysteine effectively in rheumatoid arthritis [41], but this approach is precluded where the source is infection. In IP, eradication of SIBO for several years is feasible, where adequate gastrointestinal-transit can be maintained by sufficient fluid-intake, a high-fibre diet and bulk/osmotic laxatives [18].

There is no evidence that hyperhomocysteinemia in IP is genetically determined [58]. Cognitive impairment, clinical depression, other physical incapacity and cardiovascular symptoms were exclusions [54,55]. Hyperhomocysteinemia has been attributed to levodopa: its O-methylation by catechol-O-methyltransferase (COMT) can provide a substrate for homocysteine synthesis [59]. We clearly demonstrate that the only relevance of levodopa (in modest doses) is its surrogacy for time-from-diagnosis. Moreover, neither reducing/stopping levodopa, nor inhibition of its peripheral wastage by co-prescription of a COMT-inhibitor, reduces homocysteine [59].

Anti-nuclear antibody, present in a quarter of probands, was an indicator of the outcome of anti-*Helicobacter *therapy. Outcome is uncertain in IP even when anti-microbial sensitivities are known/compliance is monitored [23,24]. Gait improves in IP following 'biopsy-proven' *H. pylori *eradication [18,23,24]. Here, following anti-*Helicobacter *therapy, gait deteriorated in the ANA-positive in stark contrast to performance in the ANA-negative. ANA appeared to alert to continuing infection, and might be more sensitive to its detection than examining two biopsies. Bolus antigen release may accentuate deterioration where anti-*Helicobacter *therapy fails. ANA-positivity and a lower CD8+ count were intrinsically linked. Reduced cytoxicity may have impaired clearance of residual organisms. Compensation by expression of CD8 on mature CD4+ cells [60] may have failed, even though an increased proportion of such cells (CD4bright+CD8dull+) has been reported in IP as a whole [5,6]. Although NK-activity is stimulated by *H. pylori in vitro *[61], the NK-count was not a prognostic indicator.

The increase, over a decade, in immunoblot-positivity over ELISA-positivity may signify more low-density and occult (gastric lymph node [62]) infection. It may or may not be IP-specific. Association of a higher lymphocyte count with immunoblot-positivity suggests that it represents infection rather than memory. More low-density infection would fit with greater incidental antimicrobial use in a context of impaired bacterial clearance. Propensity to hidden infection/resting (coccoid) forms might be greater where the 'protective' urease continues to produce ammonia in the face of cytokine-driven inhibition of gastric acid [56]. Such progressive hypochlorhydria could explain peptic ulcer being prodromal by decades in IP [26], and the increased likelihood of seropositivity in younger probands [63]. That *Helicobacter *detected only by molecular-microbiology on gastric biopsies appears to offer the same therapeutic opportunity as does frank infection [18], underlines its relevance to IP. Moreover, persistence at this level following treatment of frank infection appears detrimental [23]. These findings may predicate on an autoimmune response to *Helicobacter*.

In conclusion, the explanation of the different manifestations of IP between-probands, and within- proband over time, may lay in the interaction between more than one pathogen and determinants, genetic and environmental, of inflammatory response. *Helicobacter *may be necessary though not sufficient, SIBO a frequent, relatively non-specific and dose-related, player. A viral premonitory phase may prove the common denominator for IP, other 'neurodegenerative' diseases and neuro/psychiatric syndromes and 'functional' gastrointestinal disorders.

## Competing interests

The authors declare that they have no competing interests.

## Authors' contributions

RJD, SMD, AC, ITB, MAAI, RS and DWP designed research; RJD, SMD, ITB, MAAI, TD, AJL, AlC, NLO, JMP, JB and DWP performed research; CW contributed new analytical tools; AC,RJD, SMD and CW analyzed data; SMD, RJD, CW and AC wrote paper. All have read and approved the final manuscript.
